# Barriers and facilitators of adoption and implementation of a financial navigation program: cost-fin trial

**DOI:** 10.3332/ecancer.2026.2065

**Published:** 2026-01-22

**Authors:** Adewale Isaiah Oyewole, Funmilola Olanike Wuraola, Amir H Sohail, Titilope Ogunniyi, Chinyere Nwankwo, Zainab Oyindamola Adegbite, Dorcas Olaide Ebekue, Clement D Awe, Elizabeth N Christian, Kristina Diaz, Oluwasegun Afolaranmi, Clara N Lambert, Dan Sherman, George Gutierrez, Chinenye Iwuji, Juliet S Lumati

**Affiliations:** 1 Department of Surgery, Obafemi Awolowo University Teaching Hospitals Complex, Ife, Osun 220005, Nigeria; 2 Department of Medical Rehabilitation, Obafemi Awolowo University, Ife 220005, Nigeria; 3 Department of Surgery, Obafemi Awolowo University, Ife 220005, Nigeria; 4 Department of Surgery, University of New Mexico, Albuquerque, NM, USA; 5 Department of Oncology, Lakeshore Cancer Center, Lagos 101241, Nigeria; 6 Robert J. Havey, MD Institute for Global Health, Feinberg School of Medicine, Northwestern University, Chicago, IL 60611, USA; 7 Department of Surgery, Feinberg School of Medicine, Northwestern University, Chicago, IL 60611, USA; 8 Cancer Research UK Cambridge Institute, University of Cambridge, CB2 0RE Cambridge, UK; 9 TailorMed, New York, NY 10010, USA; 10 NaVectis Group, Grand Rapids, MI 49503, USA; 11 Center for Health Services and Outcomes Research, Feinberg School of Medicine, Northwestern University, Chicago, IL 60611, USA

**Keywords:** health care cost, resource-limited setting, literacy, financial stress, insurance coverage, treatment outcomes, developing countries, human

## Abstract

High healthcare costs are a major obstacle to treatment and recovery, particularly in low- and middle-income countries. Financial navigation programs (FNPs) are designed to help patients manage these costs by providing support through insurance and providing resources for patients. This study explores the experience of implementing FNPs in Nigeria at two tertiary cancer centers: Lakeshore Cancer Center, Lagos and Obafemi Awolowo University Teaching Hospitals Complex, Ile-Ife, Nigeria. Key barriers include low financial literacy, complex regulations and limited financial resources. However, these issues can be overcome with better financial education, simplified regulations and expanded insurance coverage. The study underscores the potential of FNPs to significantly reduce financial strain and improve treatment outcomes for cancer patients in resource-limited settings. Recommendations for the adoption and implementation of an FNP in Nigeria are included in this manuscript.

## Background

Healthcare expenses represent a significant source of stress and hardship for patients and their families [1]. Financial toxicity, a term used to describe an individual’s economic burden due to medical costs, has become a critical issue in modern healthcare systems. Patients often find themselves overwhelmed by medical bills, insurance claims and out-of-pocket (OOP) expenses. This financial strain can lead to delayed or foregone treatment, reduced adherence to prescribed therapies and diminished overall health outcomes [2].

Patient navigation programs were first introduced in the United States in the early 1990s to address inequities in healthcare access. Initially designed to help underserved populations navigate cancer care [3] these programs have since expanded to address diverse challenges, including financial toxicity. Financial Navigation Programs (FNPs) are specialised interventions to assist patients in understanding and managing the financial aspects of their healthcare [4–7]. These programs offer personalised support for navigating medical expenses, accessing available resources and understanding insurance coverage and billing processes. While FNPs have demonstrated their potential to reduce financial strain and improve patient outcomes, their adoption remains low, particularly in low- and middle-income countries (LMICs) [8, 9].

The impact of financial toxicity is particularly pronounced in LMICs, where healthcare systems are underfunded and OOP expenditures dominate medical payments [10]. Catastrophic health expenditure (CHE) is the OOP healthcare costs that significantly impact a household’s ability to maintain basic living standards, and this can lead to debt, asset liquidation or the abandonment of treatment altogether [11, 12]. For instance, a study by Knapp *et al* [12] reported a high prevalence of CHE in one of the tertiary hospitals in Nigeria. There are significant OOP costs associated with accessing breast cancer diagnosis and treatment in Nigeria. These high OOP costs resulted in over 90% of breast cancer patients at a tertiary care facility in Nigeria experiencing a CHE. CHE appears to limit access to evidence-based adjuncts (i.e., radiotherapy) and negatively impacts the well-being of the broader household [13]. In India, patients with chronic illnesses often resort to informal loans or asset sales to finance their treatment [14]. Similarly, studies in Kenya and Latin America reveal significant financial distress among patients, contributing to poor health outcomes and reduced quality of life [10, 15]. These challenges underscore the urgent need for scalable financial navigation interventions.

## Concept of FNPs

FNPs are designed to alleviate financial barriers to care by empowering patients with knowledge and resources [20]. The key components of these programs typically involve a financial assessment to evaluate patients’ financial situations and determine their ability to pay for treatments and associated costs. Resource identification is another core component, as it helps patients access financial support, such as government subsidies, charitable aid and health insurance schemes. Additionally, budgeting and planning are crucial in assisting patients with financial management to meet both current and future medical expenses. Advocacy and support are integral to the program, enabling patients to navigate complex healthcare systems and advocate for their financial needs.

FNPs aim to reduce financial distress, enhance treatment adherence and improve the overall quality of life for patients [21, 22]. A study by Yezefski *et al* [21] demonstrates that trained oncology financial navigators (FNs) significantly reduce patients’ OOP costs while also benefiting hospitals financially. That study concluded that training and deploying FNs not only lessens patients’ financial burdens but also helps healthcare systems recover costs, creating a mutually beneficial model for managing the financial toxicity of cancer care.

## Methods

### Study context

This study was conducted at two healthcare facilities in Nigeria. Lakeshore Cancer Center, a private oncology center in Lagos, is a referral hub for patients nationwide. Obafemi Awolowo University Teaching Hospitals Complex, a public tertiary healthcare institution in Ile-Ife, provides cancer care to patients referred from across Nigeria and neighbouring West African countries. These two sites cater to diverse patient populations, making them ideal for evaluating the feasibility and impact of FNPs.

### Inclusion and exclusion criteria

Patients were eligible for inclusion if they were adults (18 years and older) diagnosed with breast, prostate or colon cancer between 15 July, 2024, and 22 November, 2024, within 6 weeks prior to enrollment. Patients with recurrent cancers, those diagnosed outside the specified timeframe and children were excluded from enrollment in the study.

### Study design

This study is a single-site, pragmatic randomised controlled trial. Two FNs were hired and trained over five months by experts from NaVectis Group and TailorMed, financial navigation companies based in the United States. Their training involved one-on-one sessions with financial navigation coaches, focusing on improving patient financial navigation services at the two study locations.

In addition to assessing feasibility, the study is designed to evaluate the economic implications of financial navigation. Program costs, including training hours, salaries of navigators and resource mobilisation activities, are being tracked to inform the economic evaluation. The primary goal is the proportion of patients in each arm who experience CHE during cancer treatment at 6 a﻿nd 12 months, defined using World Bank and WHO thresholds: OOP health expenditures exceeding 10% of total household expenditure, 25% of household income or 40% of non-subsistence household expenditure. Secondary goals include: (1) Fi﻿nancial distress, measured with the validated FACIT-COST instrument administered at baseline, 3, 6 and 12 months; (2) Treatment adherence, measured as the proportion of prescribed chemotherapy, radiotherapy and surgery completed without cost-related delay or abandonment. Financial hardship is measured using an adapted financial toxicity questionnaire, combined with self-reported OOP costs and reliance on informal support networks.

To ensure the study followed the protocol, a research project manager was brought on board to oversee the work of the research assistants (Ras) and FNs. The project manager provided guidance, ensured adherence to study guidelines and facilitated communication between the Ras, FNs, data manager, data analyst, co-principal investigators and principal investigator.

### Procedures

The FNs reached out to various pharmaceutical companies, foundations, philanthropists, Health Maintenance Organisations (HMOs), the National Health Insurance Scheme (NHIS) and Non-Governmental Organisations (NGOs) to seek support for patients assigned to the financial navigation arm of the study. A total of 86 contacts were made, with 26 responses received. Of those, 14 declined to schedule a meeting for various reasons. Some foundations explained they were themselves seeking sponsorship, while others specified that their support was limited to children with cancer and not adults. The NHIS confirmed it does not cover cancer treatment expenses, and some HMOs also stated that their plans do not include such coverage. A number of pharmaceutical companies clarified that they do not produce oncology medications, and several philanthropists were unable to assist due to limited funds.

However, 12 of the 26 respondents were open to scheduling a meeting, which the FNs successfully conducted. These engagements led to eight confirmed collaborations. These included pharmaceutical companies such as Manola, Johnson & Johnson and Jehimeh, who agreed to provide 2%, 10% (for prostate drugs) and 25% discounts, respectively. Other collaborations involved cancer-focused foundations, HMOs, the PACE programme of the Medicaid Cancer Foundation and philanthropists who donated funds to support patients’ chemotherapy and surgical treatments.

All these activities began prior to the commencement of the study and are ongoing as the study progresses.

## Results

### Patient demographics

One hundred and sixteen (116) patients were recruited, and 61 were randomised to FNs and 55 into the control arm of the study. These navigators used financial literacy surveys to assess each patient’s financial literacy and developed individualised financial plans. The extent of navigation required was categorised as high (the patient had a low level of financial literacy and was not able to manage their healthcare costs independently), moderate, low or no assistance needed, with the latter indicating that the patient had a high level of financial literacy and was able to manage their healthcare costs independently. Among the 35 patients who completed financial literacy sessions, 33 received financial plans. Of these, 31 required financial navigation and 31 were successfully directed to resources, including the NHIS, philanthropic organisations, research study supports and drug discount programs.

Of the 116 patients enrolled, 65.5% were female and 34.5% were male, reflecting the inclusion of breast cancer as a predominant focus. The age distribution ranged from 32 to 87 years, with a median age of 56. Most participants resided in urban centers, reflecting a higher likelihood of access to specialised healthcare facilities. The majority of participants were married and were either self-employed or formally employed, suggesting a relatively economically active population. The majority of participants had a tertiary education, which may have implications for health literacy and understanding of medical information (Table 1).

### Socioeconomic characteristics

This summarises the socioeconomic profile of the study participants, based on income level, health insurance coverage and access to financial support (Table 2). More than half of the respondents earn less than ₦100,000 monthly, indicating that a substantial proportion of the population studied falls within the lower-income bracket. Only around one-fifth report earning ₦200,000 or more per month, highlighting potential financial vulnerability among the cohort. A significant majority of participants lack health insurance, which may imply a high reliance on OOP expenditure and increased susceptibility to financial hardship from healthcare costs. While over a quarter of participants receive no external financial support, the majority rely on relatives (43.5%) to fund treatment. Philanthropic and religious sources play a smaller but noteworthy role, contributing to 30.4% of cases combined. This underscores the importance of informal support networks in healthcare financing among this population.

## Discussion

### Barriers to financial navigation

Low financial literacy emerged as a significant barrier, with many patients lacking the knowledge necessary to make informed financial decisions. Regulatory challenges, characterised by complex and inconsistent frameworks, hindered the implementation of effective FNPs. Economic instability, marked by Nigeria’s fluctuating economy, compounded patients’ difficulties in planning and managing healthcare expenses. Communication barriers also posed challenges, as explaining financial details required significant effort from navigators, particularly for patients unfamiliar with insurance terms and funding options. Limited funding resources due to inflation and scarce philanthropic support exacerbated the difficulty of securing financial assistance for patients. Additionally, insurance limitations, including insufficient coverage under NHIS and HMOs, often resulted in substantial OOP expenses for patients. Patient refusal of financial navigation services due to perceived financial sufficiency, believing they are financially stable and do not need assistance, was also a barrier. This perception limits engagement and may overlook the broader benefits of cost optimization and resource access.

### Strategies for overcoming barriers

Implementing comprehensive financial education programs is a critical strategy for addressing low financial literacy. These initiatives aim to equip patients with the knowledge to navigate healthcare costs effectively. Regulatory reforms that streamline and simplify frameworks are essential for facilitating the adoption of FNPs. Economic stability initiatives, such as advocating for macroeconomic policies that promote stability, can enable patients to engage in better financial planning. Expanding insurance benefits to include more comprehensive cancer care services under NHIS and HMOs can significantly reduce financial burdens. Building philanthropic networks through partnerships with donors and organisations can create sustainable funding pools for patients in need. Enhancing patient awareness by simplifying enrollment processes and increasing access to financial resources is another pivotal strategy. Collaboration with government policymakers to improve healthcare affordability and accessibility is fundamental for addressing systemic challenges.

While financial navigation has been showed in high-income countries to reduce financial toxicity, the major challenge in LMICs is not its efficacy but its sustainability. Our trial emphasizes not only effectiveness but also the feasibility of long-term integration of financial navigation into Nigerian oncology services. The inclusion of a prospective economic evaluation. By documenting program startup and maintenance costs, we aim to provide evidence that will inform policymakers on the affordability and scalability of FNPs in Nigeria.

### Reflections from FNs

Navigators reported that addressing financial barriers significantly reduced patients’ stress and improved their ability to focus on treatment. By combining empathy, persistence and strategic problem-solving, navigators facilitated meaningful changes in patients’ lives, highlighting the importance of such programs in resource-limited settings [16–19].

## Conclusion

Preliminary findings from this study highlight the potential of FNPs to alleviate financial toxicity and improve treatment adherence among cancer patients in Nigeria. Importantly, by adding measures of both patient outcomes and program costs, this study addresses the sustainability and scalability of such interventions in resource-limited settings. Addressing barriers such as low financial literacy, limited resources and systemic regulatory challenges is essential for scaling these programs. Future efforts should focus on refining program components, fostering collaborations with stakeholders and advocating for policies that promote equitable access to healthcare.

## Recommendations

1. Partnerships with local NGOs: Collaborating with local NGOs that have a presence in underserved areas can extend the reach of financial navigation services. NGOs often have community ties and resources that complement FNPs.
2. Capacity building in healthcare facilities: Training healthcare staff to serve as FNs should be a priority. Establishing resource-sharing agreements with healthcare facilities (e.g., data sharing or joint staffing) can alleviate resource constraints.
3. Simplified communication strategies: FNs should be trained to communicate complex information in simple, understandable language. Educational materials like brochures, videos and infographics should be adapted to local languages and literacy levels.
4. Cultural sensitivity training for navigators: FNs should receive cultural competence training to approach discussions of financial difficulties with empathy. They should also be educated about local cultural norms to better connect with patients.
5. Confidential and private counseling: Providing private, confidential spaces for financial consultations will encourage patients to speak openly about their financial situations without fear of judgment or exposure.
6. Integration with health insurance providers: FNPs can integrate with health insurance providers to help patients understand their benefits and coverage. Insurance companies can partner with FNPs to provide navigators with access to up-to-date coverage details.
7. Creation of centralised financial assistance databases: Developing a centralised database of financial aid programs can help FNs quickly identify and apply for assistance. Governments and NGOs can collaborate to maintain such databases.
8. Diversified funding models: FNPs should diversify funding by seeking support from government health budgets, private foundations, health insurance companies and international donors. This reduces dependency on a single funding source.
9. Policy advocacy for insurance reforms: Advocacy efforts should focus on expanding health insurance coverage, simplifying enrollment processes and integrating financial navigation into insurance systems. Collaboration with policymakers can streamline insurance schemes.

## Conflicts of interest

The authors declared no conflicts of interest.

## Author contributions

All the authors reviewed this manuscript.

## Trial registration

CMUL/HREC/02/25/1392/V1 and ERC/2024/05/12/ IRB/IEC/0004553.

## Figures and Tables

**Table 1.** Demographic characteristics.

Variables	Criteria	Control (RC) *N* = 55	Treatment (FN) *N* = 61	*p*-value
Age (years)	25–39	4 (7.4%)	7 (11%)	0.2
	40–69	41 (76%)	36 (59%)	
	≥70	9 (17%)	18 (30%)	
Gender	Male	17 (31%)	22 (36%)	0.7
	Female	37 (69%)	39 (64%)	
Marital status	Married	42 (76%)	52 (85%)	0.2
	Formerly married	13 (24%)	9 (15%)	
Education	None	7 (13%)	5 (8.5%)	0.9
	Primary	8 (15%)	10 (17%)	
	Secondary	16 (30%)	20 (34%)	
	Tertiary	22 (42%)	24 (41%)	

**Table 2.** Socioeconomic characteristics.

Variables	Criteria	Control (RC)	Treatment (FN)	*P*-value
Household size	Median #	4 (SD ±2)	4 (SD ±2)	0.4
Median monthly household income	₦ (Naira)	67,500 (7,000–800,000)	71,000 (5,000–800,000)	0.3
Household wealth quintiles	Very Poor	28 (53%)	35 (57%)	0.4
	Poor	7 (13%)	12 (20%)	
	Middle	6 (11%)	8 (13%)	
	Rich	9 (17%)	5 (8.2%)	
	Very Rich	3 (5.7%)	1 (1.6%)	
Employment status	Employed	22 (49%)	18 (37%)	0.5
	Unemployed	10 (22%)	12 (24%)	
	Retired	13 (29%)	19 (39%)	
Health insurance	Insured	11 (21%)	7 (11%)	0.2
	uninsured	42 (79%)	54 (89%)	

## References

[1] Carrera PM, Kantarjian HM, Blinder VS (2018). The financial burden and distress of patients with cancer: understanding and stepping‐up action on the financial toxicity of cancer treatment. CA A Cancer J For Clinicians.

[2] Zafar SY, Abernethy AP (2013). Financial toxicity, Part I: a new name for a growing problem. Oncology.

[3] Freeman H.P (2006). Patient navigation: a community-based strategy to reduce cancer disparities. Cancer Epidemiol Biomark Prev.

[4] Zullig LL, Peppercorn JM, Schrag D (2013). Financial distress, use of cost-coping strategies, and adherence to prescription medication among patients with cancer. J Oncol Pract.

[5] Vipond TA (2018). (Doctoral dissertation, Bradley: Bradley University). Improving Healthcare Financial Literacy in Nurse Practitioners with Concise, Applicable Healthcare-Focused Financial Education.

[6] Doherty MJ, Thom B, Gany F (2021). Evidence of the feasibility and preliminary efficacy of oncology financial navigation: a scoping review. Cancer Epidemiology Biomarkers & Prevention.

[7] Linendoll N, Murphy-Banks R, Sae-Hau M (2023). Evaluating the role of financial navigation in alleviating financial distress among young adults with a history of blood cancer: a hybrid type 2 randomized effectiveness-implementation design. Contemp Clin Trials.

[8] Banegas MP, Dickerson JF, Friedman NL (2019). Evaluation of a novel financial navigator pilot to address patient concerns about medical care costs. Permanente J.

[9] Watabayashi K, Steelquist J, Overstreet KA (2020). A pilot study of a comprehensive financial navigation program in patients with cancer and caregivers. J Nat Comprehensive Cancer Netw.

[10] Knaul FM, Farmer PE, Krakauer EL (2015). Alleviating the access abyss in palliative care and pain relief—an imperative of universal health coverage: the Lancet Commission report. Lancet.

[11] Onwujekwe O, Hanson K, Uzochukwu B (2012). Examining inequities in incidence of catastrophic health expenditures on different healthcare services and health facilities in Nigeria. PLoS One.

[12] Knapp GC, Wuraola FO, Olasehinde O (2022). The out-of-pocket cost of breast cancer care at a public tertiary care hospital in Nigeria: an exploratory analysis. Pan Afr Med J.

[13] Wuraola FO, Blackman C, Olasehinde O (2024). The out-of-pocket cost of breast cancer care in Nigeria: a prospective analysis. J Cancer Policy.

[14] Pramesh CS, Badwe RA, Sinha RK (2014). Financial toxicity of cancer treatment: moving the discourse from acknowledgment to action. Lancet Oncol.

[15] Gathu KE, Muthuri R, Mutiso R (2018). Financial distress in cancer care: perspectives from Kenya. BMC Health ServRes.

[16] Dohan D, Schrag D (2005). Using navigators to improve care of underserved patients: current practices and approaches. Cancer.

[17] Hendren S, Griggs JJ, Epstein RM (2010). Study protocol: a randomized controlled trial of patient navigation-activation to reduce cancer health disparities. BMC Cancer.

[18] Natale‐Pereira A, Enard KR, Nevarez L (2011). The role of patient navigators in eliminating health disparities. Cancer.

[19] Budde H, Williams GA, Scarpetti G (2022). What are patient navigators and how can they improve integration of care?.

[20] Gabitova G, Burke NJ (2014). Improving healthcare empowerment through breast cancer patient navigation: a mixed methods evaluation in a safety-net setting. BMC Health ServRes.

[21] Yezefski T, Steelquist J, Watabayashi K (2018). Impact of trained oncology financial navigators on patient out-of-pocket spending. Am J Manag Care.

[22] NaVectis (n.d.) Home.

